# Lifefulness - feasibility and acceptability of a self-guided digital intervention promoting mental health among older adults in Sweden^☆^

**DOI:** 10.1016/j.invent.2026.101005

**Published:** 2026-09-28

**Authors:** Martin Kraepelien, Karin Romberg, Neda Agahi, Nitya Jayaram-Lindström, Amit Chakrabarti, Arun Kandasamy, Indranil Saha, Christopher Sundström

**Affiliations:** a Centre for Psychiatry Research, Department of Clinical Neuroscience, Karolinska Institutet and Stockholm Health Care Services, Region Stockholm, Sweden; b Aging Research Center, Department of Neurobiology, Care Sciences and Society, Karolinska Institutet, Stockholm, Sweden; c Department of Mental Health, ICMR-Centre for Ageing and Mental Health, Indian Council of Medical Research, Kolkata, West Bengal, India; d AOD and Gaming Disorder services, SMHS - Fiona Stanley Hospital, Murdoch, Western Australia, Australia; e ICMR-National Institute for Research in Bacterial Infections, Indian Council of Medical Research, Kolkata, West Bengal, India; f Department of Psychology, Uppsala University, Uppsala, Sweden; g Stockholm Health Care Services, Region Stockholm, Sweden

**Keywords:** Self-guided intervention, Digital intervention, Older adults, Healthy ageing, Mental health, Feasibility trial

## Abstract

**Background:**

The number and proportion of older adults (≥65 years) is increasing worldwide, already exceeding 20% in Sweden. The growing number of older adults with concurrent physical and mental health issues, pose a major challenge for healthcare, rendering a critical need for low-cost, easily accessible behavioural interventions.

**Methods:**

This single-group feasibility trial evaluated a transdiagnostic digital self-help intervention, “Lifefulness”, specifically adapted for older adults and designed to promote behaviour change and mental health. Content was based on cognitive behavioural therapy (CBT) and healthy ageing principles. Older adults were recruited from the community (age range 65–85) and were assigned to the six week intervention, which included three brief phone interviews focusing on program adherence. Outcomes included program use and acceptability as well as preliminary effects on quality of life and mental health outcomes.

**Results:**

A total of 73 individuals were included. Program utility was high on average, with 72% of participants engaging with the intervention daily or several times a week. The average time spent on the three interviews was 33 min per participant. The program was associated with favourable changes in various mental health symptoms and higher ratings of quality of life and the participants reported high satisfaction and credibility.

**Conclusions:**

These findings support the feasibility and resource-efficiency of a digital self-help intervention for older adults. The intervention warrants further testing in a randomized trial with long-term follow-up assessments of both outcomes and healthcare consumption.

## Highlights

• A digital self-help program was feasible for older adults aged 65–85
• Participants showed high engagement, with 72% using the program several times a week
• Clinician time spent averaged 33 min per participant
• The program was associated with improved mental health and quality of life
• Results support further testing in randomized trials

## 1 Background

### 1.1 Introduction

Societies worldwide are experiencing a demographic shift marked by a growing proportion of older adults (Gu et al., 2021). In Sweden, older adults (≥65 years) currently constitute 20% of the population, a proportion projected to increase further in the coming decades (SCB, n.d.). This growing demographic, often characterized by the presence of medical comorbidities and concurrent mental health issues, presents a significant and escalating challenge to healthcare systems and public welfare (Reynolds et al., 2022).

A robust body of evidence demonstrates that digital interventions based on cognitive behavioural therapy (CBT) are effective in supporting individuals to successfully manage mental health problems (Löchner et al., 2025). Such interventions may therefore be well positioned to play a significant role in the future of mental health care also for older adults. Although internet use remains lower in this age group, this digital gap is shrinking and is expected to vanish with future cohorts (Hunsaker and Hargittai, 2018). Qualitative evidence further suggests that older adults are generally receptive to digital interventions, while highlighting the importance of age-appropriate design and access to human support (Yin et al., 2024). While digital interventions promoting physical activity and nutrition among older adults show some efficacy (Buyl et al., 2020), most mental health research has focused on younger or middle-aged adults. A 2022 review identified only eleven relevant studies in this population, primarily targeting depression and anxiety (Dworschak et al., 2022). More recent systematic reviews demonstrate that the literature is expanding rapidly, indicating favourable effects of digital interventions on depression and anxiety in older cohorts (Shi et al., 2024; Ibrahim et al., 2026). However, both of these reviews adopted broad age criteria, including individuals from age 50, meaning that evidence specifically targeting older populations (≥65 years) remains comparatively sparse. In addition, these reviews predominantly evaluated digital interventions outside structured psychological frameworks, such as exergames, stepping devices, and wearable sensors. In contrast, Swedish randomized trials have shown that tailored internet-delivered CBT can successfully reduce depression and anxiety in older adults (Silfvernagel et al., 2018; Nordgren et al., 2024), but these interventions have typically focused on clinician-guided treatment for specific disorders. Advancement in the field is urgent, considering that psychopharmacological treatments (e.g., antidepressants, benzodiazepines) are often contraindicated in older adults due to the high prevalence of multimorbidity and potential drug-drug interactions (Kok and Reynolds, 2017; Markota et al., 2016). Addressing this need requires the development of low-cost, easily accessible interventions that promote healthy behaviours, improve mental health outcomes, and reduce the burden on health care. Mental health difficulties among older adults do not always meet diagnostic thresholds, and subthreshold symptoms of depression and anxiety are common in this population (Meeks et al., 2011; Witlox et al., 2021), symptoms which nevertheless affect well-being and functioning and constitute an important target for preventive interventions. Whereas existing digital programs for older adults typically focus either on healthy ageing and lifestyle promotion (Tsai et al., 2024) or on discrete psychiatric diagnoses or conditions (Riadi et al., 2022; Shah et al., 2021), there is a need for broader preventive interventions that address the interconnected psychological, social, and behavioural challenges that commonly arise in later life. Low-cost, scalable interventions may be used as digital health promotion (Stark et al., 2022) or indicated prevention (Ebert et al., 2017) of future mental health issues and healthcare use. To address this gap, we developed the digital program Lifefulness to target everyday determinants of well-being while promoting meaning and participation in later life. However, evaluating user engagement and procedural feasibility is essential before testing such novel, low-threshold frameworks at scale.

### 1.2 Aims

The objectives of the current study were to examine the feasibility and acceptability of a new digital intervention, Lifefulness, promoting mental health among older adults in Sweden, utilizing metrics such as program use and participant perceptions. Further aims included assessing preliminary effects on health-related quality of life and mental health symptoms, as well as quantifying resource consumption through clinician time spent per participant on supporting phone interviews.

## 2 Methods

### 2.1 Trial setting

This was a single-group open trial, conducted at the Centre for Psychiatry Research, Stockholm Health Care Services and Karolinska Institutet, Sweden, from April to October 2025. The study received approval from the Swedish Ethical Review Authority (2024-00363-01, amended 2025-01276-02).

### 2.2 Patient and public involvement

Patient and public involvement was integral to the development and refinement of the intervention. Prior to development, interview sessions were conducted with clinicians and researchers in the Stockholm region specializing in older adults to inform the program's overall approach. Subsequently, a total of seven focus groups were held, each consisting of two sessions and comprising between three and five older adult participants. One focus group included both genders, while the remaining six were gender-divided into three groups of men and three groups of women. The first session focused on gathering participants' general views on healthy ageing, mental health, and the use of digital tools and interventions. The qualitative results from these initial groups will be presented in a forthcoming separate paper. The second session was conducted after participants had tested an early version of the intervention. During this session, participants provided direct feedback and suggestions on the program's content, tone, exercises, and examples. Their input was used specifically to inform language refinements and to improve the overall relevance and clarity of the program materials.

### 2.3 Participants

Eligible participants were individuals aged 65 to 85 years, proficient in Swedish, with access to an internet-connected device, and with an interest in promoting their mental health. Participants were excluded if they presented with an ongoing unstable psychiatric condition requiring inpatient care or intensive psychiatric treatment; severe suicidal ideation; active, untreated mania, psychosis, or cognitive impairment. Further exclusion criteria included having a neurological disorder (e.g., dementia, severe Parkinson's disease, multiple sclerosis) or physical impairment that would interfere with participation. Individuals concurrently participating in another psychological intervention study were also excluded.

### 2.4 Procedure

Recruitment of participants was conducted via advertisements placed on social media platforms (Facebook and Instagram). Interested individuals registered on a secure website where they provided digital informed consent and contact information, and completed an initial online screening assessment. Prospective participants who met preliminary criteria were contacted for a telephone screening interview, which also served as initial onboarding to the intervention. Exclusion criteria were assessed by the clinical psychologist during the interview to ensure participant safety and suitability for the intervention. Individuals deemed to require more intensive treatment were referred to adequate care. Enrolled participants were notified and granted access to the digital intervention the following Monday. The digital intervention ran for six weeks. Online questionnaires were administered during the screening procedure which acted as baseline, at the mid-point (Week 3), at the post-intervention follow-up (Week 6), and lastly at a three-month follow-up (3 months after the end of the intervention).

### 2.5 Intervention

The Lifefulness program is based on principles in CBT and healthy ageing and consists of six modules addressing common mental health challenges in later life, including loneliness, anxiety, sleep difficulties and unhealthy habits (see Table 1 for an overview of the modules). The first module introduced the two concepts healthy ageing and lifefulness. The concept of lifefulness (“livsfullhet” in Swedish) was developed within the project in collaboration with focus group participants and was used to capture a broad, positive view of ageing that extends beyond the absence of mental health problems. It refers to a sense of meaning, engagement, vitality, and continued participation in life, including the capacity to experience life as rich and worthwhile despite age-related changes or limitations. Theoretically, the program is informed by a lifespan developmental perspective, viewing psychological well-being as an adaptive process rather than merely the absence of symptoms (Baltes et al., 2007). In adapting standard behavioural activation for older cohorts, principles from the Selection-Optimization-Compensation model (Baltes and Carstensen, 2003) were utilized to guide participants in prioritizing meaningful goals (selection), optimizing personal resources, and adopting compensatory strategies when facing physical or functional constraints. Accumulating evidence suggests that wisdom and its core components – such as compassion, emotional regulation, and acceptance of uncertainty – are inversely associated with depressive symptoms and may function as protective factors in late life (Jeste et al., 2025). Behavioural activation is a promising and easy to understand CBT-method, that can reduce depression and increase healthy behaviours in older adults (Polenick and Flora, 2013). Each subsequent module follows a distinct theme relevant to older adults, including sleep problems (Jaussent et al., 2011), loneliness (Ong et al., 2016), worry and anxiety (Wolitzky-Taylor et al., 2010), and unhealthy habits such as alcohol consumption (Raninen and Agahi, 2020). Each module combines psychoeducation, practical exercises, and reflections designed to be accessible and meaningful for older adults. A special feature of the program is its narrative design: participants follow five fictional characters throughout the intervention. These characters reflect different life situations, challenges, common transitions such as retirement (Wang and Shi, 2014) and bereavement (Naef et al., 2013), and coping strategies relevant to older adults, and were co-created together with the focus groups to ensure authenticity and resonance. In line with socioemotional selectivity theory (Carstensen, 2006), which posits that older adults prioritize emotionally meaningful goals and social connections over informational acquisition, the narratives emphasized emotionally salient themes, valued relationships, and adaptive coping rather than purely instructional content. The narratives provide a relatable and engaging framework, helping participants recognize themselves and apply the intervention's strategies in their own lives. In addition to the weekly modules and the fictional characters, the program includes a digital self-help tool, similar to earlier intervention studies conducted by the research group (Kraepelien et al., 2023a). In the current study, this central tool was called *the lifefulness diary*, a simple activity diary based on behavioural activation for depression, and tailored specifically for older adults to register and track daily target behaviours as well as reflect on the concept of lifefulness. Emphasizing positive aspects of ageing - such as resilience, wisdom, and prosocial engagement - is essential both to counter stigma and to strengthen mental health in later life (Jeste et al., 2025). Therefore, the intervention not only addresses common mental health challenges but also actively supports healthy ageing and living in line with personal values. In this way, the intervention reflects the growing evidence base for a *positive psychiatry of ageing*, highlighting that well-being and growth remain possible through later life (Löchner et al., 2025).

**Table 1:** Overview of phone interviews, and weeks with self-help content and word count.

Component	Description	Word count mandatory texts	Word count optional texts
Initial phone interview	In addition to study inclusion, this call focused on program information, onboarding, motivation, and addressing any questions about participation.	–	–
Lifefulness diary	The digital tool for behavioural activation adapted for older adults focusing on increasing physical, social, and mentally stimulating activities. Used during six weeks.	114 words	–
Module 1: Lifefulness and healthy ageing	Introduction to the program, its example characters, and their stories. The module also covers the concept of lifefulness, behaviours associated with healthy ageing, and instructions on how to use the lifefulness diary.	6876 words	711 words
Module 2: Sleep problems	Strategies for sleep hygiene and sleep restriction are introduced. Continued work with the diary and reading about the characters' progress.	4942 words	232 words
Module 3: Loneliness	Strategies to increase social interaction and challenge negative automatic thoughts are introduced. Continued work with diary and characters' stories.	3991 words	193 words
Mid intervention phone interview	The second call focused on the participant's activity level in the program, with the interviewer aiming to highlight a positive observation. Help was provided if the participant had difficulties with engagement.	–	–
Module 4: Worry and anxiety	Strategies including how to challenge anxious thoughts and emotions are introduced. Continued work with diary and characters' stories.	3677 words	126 words
Module 5: Unhealthy habits	Strategies to explore motivation for change, identify risk situations, and urge surfing are introduced. Continued work with diary and characters' stories.	4518 words	107 words
Module 6: Summary and review	Theme: Summary and future. Continued work with diary and characters' stories.	3192 words	72 words
Post intervention phone interview	The third call was aimed at discussing the participant's program engagement and providing feedback. The interviewer also encouraged the participant to continue working with the strategies and behaviours that had been effective.	–	–

The user interface of the digital intervention was designed to be user-friendly, employing a mobile-first design approach with automated features and a stepwise presentation in small chunks. This design was implemented as it may facilitate higher levels of behavioural engagement compared to more basic user interfaces (Hentati et al., 2021; Hentati et al., 2025). A screenshot of the graphical user interface is available in Fig. 1. An important component of the intervention was the brief telephone interviews made at the mid-point and post-intervention. Furthermore, automated text message reminders, including a link to the intervention login, were sent twice weekly. The total word count of the intervention was 27,310 words, plus 1441 optional words (Q&A), totalling 28,751 words. Within the context of digital CBT-based self-help interventions in Sweden, this word count is more condensed compared to traditional interventions, which may use approximately 60,000 words (Karlsson-Good et al., 2023). An overview of the phone interviews, weekly content, and word count is provided in Table 1.

**Fig. 1:**
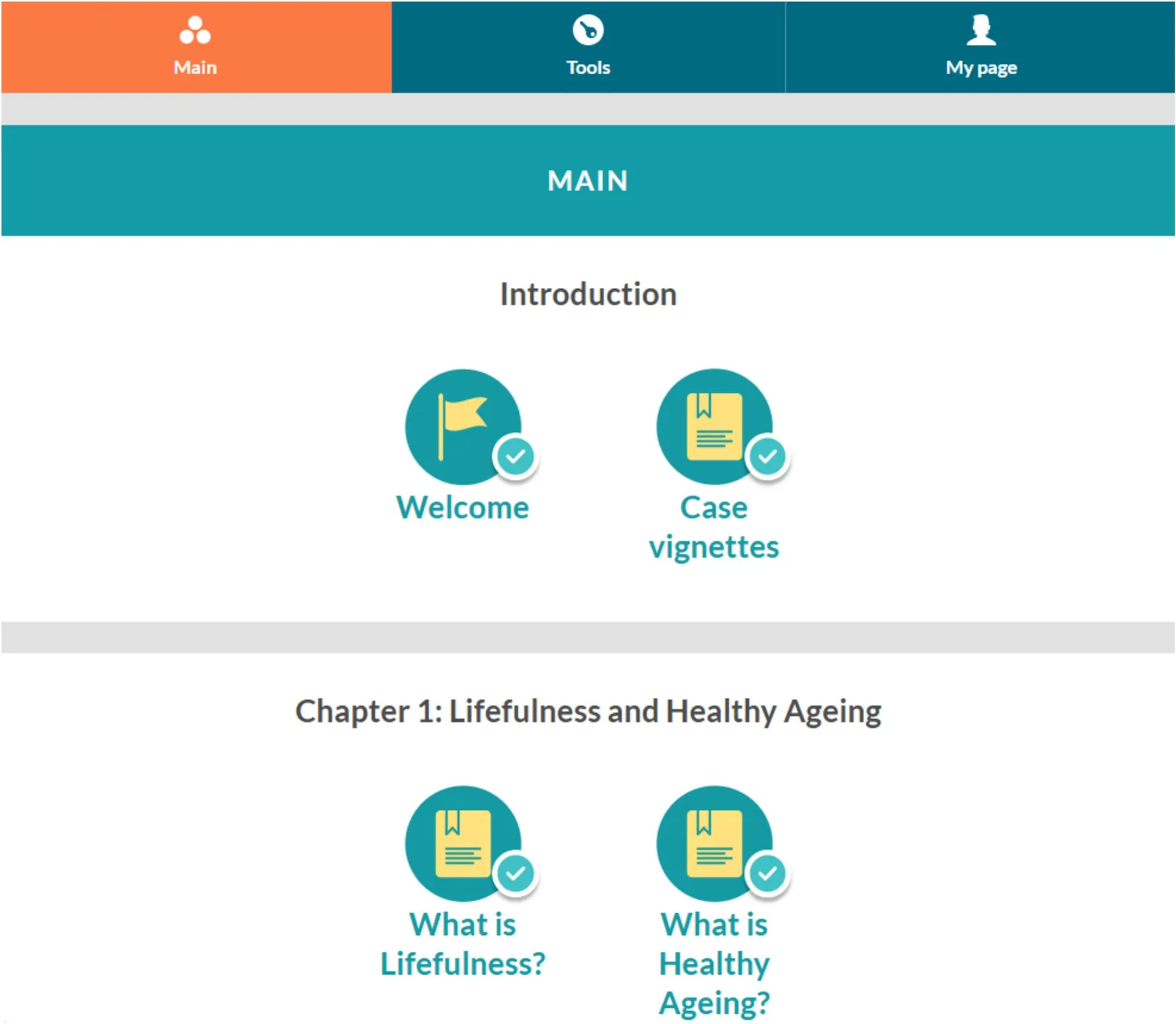
Screenshot of the graphical user interface, translated to English.

### 2.6 Measures

#### 2.6.1 Feasibility, engagement and acceptability

To distinguish between study feasibility, program engagement, and acceptability, these outcomes were evaluated separately. *Feasibility* and *engagement* were assessed on an exploratory basis using both objective engagement metrics and a self-rated measure of use, evaluated descriptively without prespecified criteria. Objective metrics included the number of completed modules and the number of diary registrations in the lifefulness diary. These were supplemented by a self-rated question administered post-intervention regarding frequency of overall intervention use. Response options for the self-rated measure were: *Daily or almost daily, Several times a week, Around once a week*, or *Never or almost never*.

*Acceptability* was measured at the post-intervention assessment using four specific questions, previously used by the research group in a similar study (Kraepelien et al., 2023b), where participants rated whether: 1) the intervention was likable, 2) the intervention was easy to understand, 3) the example narratives felt relevant, and 4) functionality and information contributed to the participant feeling overwhelmed. These four questions were answered on a four-point scale ranging from 0 (strongly disagree) to 3 (strongly agree); however, the scale for the Overwhelmed-item was reversed, with scores ranging from 3 (strongly disagree) to 0 (strongly agree), reflecting being overwhelmed as a negative outcome. A mean score above 1.5 was considered to indicate a generally positive attitude across all four items, in line with previous feasibility evaluations using these questions (Kraepelien et al., 2023b). In addition to these quantitative ratings, qualitative quotes were collected via opportunities for participants to provide free-text feedback on both the intervention and the lifefulness diary within the platform (n = 68). Furthermore, during the post-intervention phone interview (n = 60), participants were specifically asked about their future care needs. Selected quotes were chosen to illustrate the full range of positive and negative feedback provided, alongside statements representing typical needs regarding future healthcare consumption. The quotes serve purely as descriptive illustrations to contextualise the quantitative acceptability ratings. A comprehensive qualitative analysis of participant experiences will be reported separately.

*Intervention credibility* was measured during the intervention (after 2 weeks) with a five-item version of the **Credibility/Expectancy Questionnaire** (Borkovec and Nau, 1972). The scale consists of five 11-point items (0–10), yielding a score of 0–50 points in total, with a higher score reflecting higher treatment credibility. A total score of 30 points or higher is considered to indicate adequate treatment credibility.

*Intervention satisfaction* was measured post-intervention with the **Client Satisfaction Questionnaire-8** item version (CSQ-8) which ranges from 8 to 32 points (Attkisson and Zwick, 1982). A score of 8–13 was considered poor, 14–19 was considered fair, 20–25 was considered good, and 26–32 was considered excellent (Smith et al., 2014).

*Resource consumption*, which serves as a key measure of feasibility, was quantified by logging the total time the study psychologists spent on the phone interviews with each participant.

#### 2.6.2 Clinical and other measures

The **36-Item Short Form Health Survey (SF-36)** was the primary outcome and was administered at baseline, at mid-intervention, at post-intervention and at the three-month follow-up. As a practical and valid instrument for administration to older adults (Walters et al., 2001), it was used to measure health-related quality of life across eight distinct subscales, which are subsequently aggregated into two main components: the Physical Component Summary (PCS) and the Mental Component Summary (MCS). Component summary raw scores were used to evaluate within-subject change across the four time points (Ware et al., n.d.). General subjective quality of life was assessed using the Visual Analogue Scale (VAS) from the **EuroQOL (EQ-5D**) (Herdman et al., 2011), administered at both baseline and post-intervention assessments. Depressive symptoms were measured at baseline, mid-point, post-intervention, and follow-up using the **Geriatric Depression Scale** (GDS-15) (Yesavage and Sheikh, 1986). Anxiety symptoms were measured at baseline, mid-point, post-intervention, and follow-up using the **Geriatric Anxiety Scale** (GAS-10) (Mueller et al., 2015). Suicidal ideation was measured at baseline, mid-point, post-intervention, and follow-up using Item 9 of the Montgomery-Åsberg Depression Rating Scale–Self-Rated (MADRS-S Item 9) (Svanborg and Åsberg, 2001). Loneliness was measured at baseline and post-intervention using the **6-item De Jong Gierveld Loneliness Scale** (DeJong) (Gierveld and Tilburg, 2006). Insomnia symptoms were measured at baseline, mid-point, post-intervention, and follow-up using the **Insomnia Severity Index** (ISI) (Bastien et al., 2001). Alcohol problems were measured at baseline, mid-point, post-intervention, and follow-up using the **Alcohol Use Disorders Identification Test** (AUDIT) (Saunders et al., 1993). A cut-off adapted for older adults of ≥5 for men and ≥4 for women (Van Gils et al., 2021) was used. Physical activity was assessed at baseline and post-intervention using the **International Physical Activity Questionnaire** (IPAQ) (Craig et al., 2003). Due to the IPAQ's known issues with over-reporting and difficulties in quantifying time within the older population (Grimm et al., 2012), a custom dichotomization was employed. This approach focused on the proportion of participants who reported ≥3 days of moderate or vigorous physical activity during a week, based on the lowest frequency target for vigorous activity in the IPAQ's scoring protocol. This was utilized as a proxy measure for regular activity. Specifically, the 3-day threshold aligns with the minimum weekly frequency benchmark required for moderate-to-high activity categories in the official IPAQ scoring protocol, while avoiding unreliable duration estimates. Finally, the **Perceived Ageism Questionnaire (PAQ-8)** (Brinkhof et al., 2022) was used to assess ageism. PAQ-8 is a relatively new scale with research still emerging, so there are no established cut-offs in the literature yet for mild, moderate and severe perceived ageism. The PAQ-8 uses separate positive and negative subscales. The range of scoring is 8–40, with subscale ranges of 3–15 for positive and 5–23 for negative ageism. The PAQ-8 was administered exclusively at baseline to assess levels of positive and negative ageism present in the study sample.

### 2.7 Statistical considerations

As the primary objective of this trial was to assess the program's feasibility and acceptability, the study was not formally powered for hypothesis testing on clinical outcomes (Lakens, 2022). Instead, we aimed to recruit a minimum of approximately 75 older adults. This sample size was anticipated to be sufficient for a robust evaluation of feasibility and to ensure the inclusion of participants representing a broad, qualitative variety of experiences, ages, and socio-economic statuses.

Data on feasibility, acceptability, credibility, satisfaction as well as all clinical outcomes were analysed using descriptive statistics and within-group effect sizes with 95% confidence intervals using the participants with complete data at the post (n = 68) and three month follow-up (n = 62) time points. Following confirmation of normal distribution, paired samples *t*-tests were used to assess within-group changes for statistical significance. Exploratory post-hoc analyses were performed that included separate analyses for the Mental Component Summary (MCS) of the SF-36 broken down by age and gender, as well as mental health outcomes for participants who scored above relevant clinical cut-offs at baseline. Further, a Pearson's correlation analysis (r) was performed between objective adherence metrics (number of diary registrations and completed modules) and the change in MCS raw score to examine potential dose-response relationships. Statistical analyses were conducted using Jamovi (Version 2.3) and R (Version 4.1).

## 3 Results

### 3.1 Sample characteristics and study flow

Out of the 108 individuals who signed up for the study, 73 were included between April 24 and May 9, 2025. The study flow and data attrition are presented in the flow chart (see Fig. 2). The majority of participants were female, aged between 65 and 74 years old, had a partner, a high level of education, and reported having a good financial situation (see Table 2). All participants except ten (14%) self-reported that they had at least one problem area of the four problem areas with corresponding in-depth modules (sleep problems, loneliness, worry and anxiety, and unhealthy habits). The most common problem area was sleep problems, followed by unhealthy habits, worry and lastly loneliness. The participants reported much higher levels of positive ageism compared to negative ageism (Table 2).

**Fig. 2:**
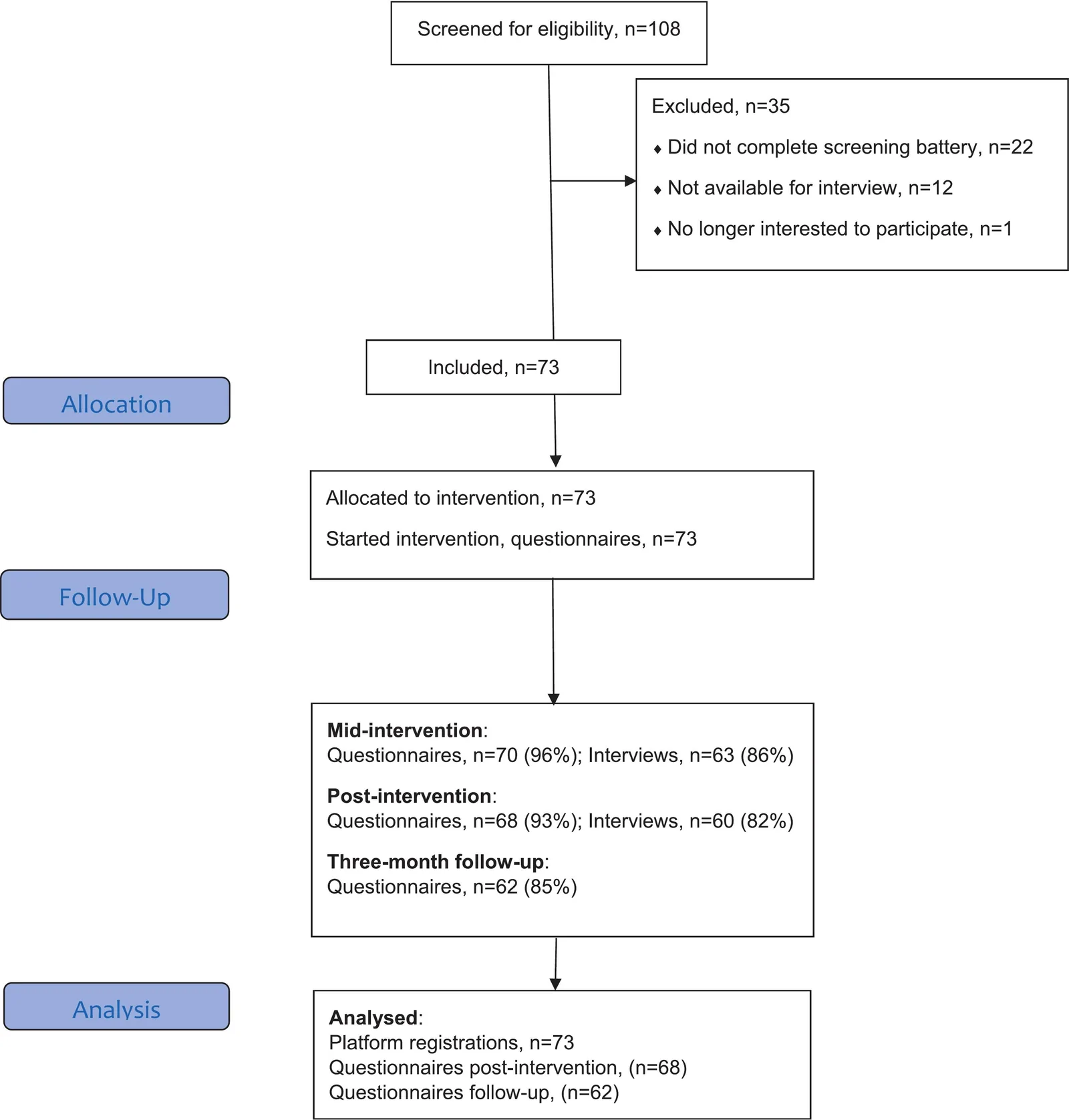
Study flow chart.

**Table 2:** Participant characteristics at screening (n = 73).

Variable	
Female, n (%)	52 (71%)
Age, mean (range)	70.7 (65–85)
Proportion 65–74 years, n (%)	56 (77%)
Proportion 75–85 years, n (%)	17 (23%)
Highest level of education, n (%)	
Elementary school	1 (1%)
Senior High School	19 (26%)
University	51 (70%)
Other	2 (3%)
Marital status, n (%)	
Married or partner	45 (62%)
Divorced	8 (11%)
Widow or widower	7 (10%)
Single or other	13 (18%)
Current occupation status, n (%)	
100%	5 (7%)
75%	2 (3%)
50%	4 (6%)
25%	19 (26%)
0%	43 (59%)
Current self-reported financial situation, n (%)	
Very poor	1 (2%)
Poor	7 (10%)
Neither poor or good	16 (22%)
Good	35 (48%)
Very good	14 (19%)
Self-reported problem areas^a^, n (%)	
Sleep-problems	41 (56%)
Involuntary loneliness	15 (21%)
Worry or anxiety	27 (37%)
Unhealthy habits	29 (40%)
Perceived Ageism, PAQ-8 total score, mean (SD)	16.6 (3.4)
Positive ageism sub score (range 3–15)	8.8 (2.6)
Negative ageism sub score (range 5–23)	7.8 (2.9)

a Self-reported problem areas may overlap; PAQ-8, Perceived Ageism Questionnaire.

### 3.2 Feasibility, engagement and acceptability

Module completion was high among the 73 participants. Five participants (7%) did not complete any modules, 43/73 (59%) completed all six modules, and 18/73 (25%) completed most modules (more than half, but not all). In summary, 61/73 (84%) of participants completed most or all modules. The mean completion rate across the entire sample was 4.9 out of 6 modules, corresponding to an average completion of 82% of the intervention. Diary registration compliance, was measured over the six-week intervention period (maximum 42 possible registered days). Four participants (5%) made no diary entries, 22/73 (30%) registered entries on all 42 days, and an additional 32/73 (44%) registered entries on most, but not all, days. In summary, 54/73 (74%) of participants registered most or all possible diary entries. The mean number of 29.5 out of 42 possible diary registrations corresponds to an average usage of 70% of all days during the intervention. These registration figures align with the participants' self-rated use of the intervention, where 49/68 (72%) reported using the intervention daily, almost daily, or several times a week.

Regarding acceptability thresholds, participants reported high ratings for satisfaction and credibility, and found the intervention likable, easy to understand, not overwhelming and that it contained relevant examples (see Table 3). The total time spent by clinicians per participant averaged just over half an hour, distributed across three scheduled telephone interviews: one before, one during, and one after the intervention period. To illustrate the variety of experiences, perceptions and needs reported by participants, selected quotes encompassing both positive and negative feedback, as well as future needs of healthcare are provided in Textbox 1. Most participants reported high satisfaction with the intervention and indicated no current need for further care. Table 3 presents adherence and engagement metrics, including the number of modules completed, diary entries, self-rated use, and clinician time spent per participant, alongside participants' perceptions of the intervention's usefulness and credibility.

**Table 3:** Number of modules, diary entries, self-rated use, perceptions, satisfaction and credibility of intervention, as well as clinician time spent per participant.

Variable	n (% completed)	Result
Number of completed modules (0–6), m (SD)	73 (100%)	4.9 (1.8)
Number of diary registrations (0–42), m (SD)	73 (100%)	29.5 (15.2)
Self-rated frequency of use of intervention	68 (93%)	
Daily or almost daily, n (%)		22/68 (32%)
Several times a week, n (%)		27/68 (40%)
Around once a week, n (%)		15/68 (22%)
Never or almost never, n (%)		4/68 (6%)
Self-rated perceptions of intervention (scale 0–3)	66 (90%)	
Likable, m (SD) [95% CI]		2.2 (0.5) [2.0–2.3]
Easy to understand, m (SD) [95% CI]		2.4 (0.5) [2.2–2.5]
Relevant examples, m (SD) [95% CI]		1.9 (0.6) [1.7–2.0]
Feeling overwhelmed^a^, m (SD) [95% CI]		2.0 (0.8) [1.8–2.2]
Satisfaction, CSQ-8 (8–32), m (SD) [95% CI]	66 (90%)	24.2 (4.4) [23.2–25.3]
Proportion ≥ 18, n (%)		61/66 (92%)
Credibility, CEQ (0–50), m (SD) [95% CI]	70 (96%)	32.0 (7.8) [30.1–33.8]
Proportion ≥ 31, n (%)		48/70 (69%)
Clinician time per participant in minutes	73 (100%)	
Initial phone interview, m, median (min–max)		13.7, 13 (5–30)
Mid phone interview, m, median (min–max)		10.5, 8 (0–47)
Post phone interview, m, median (min–max)		8.9, 7 (0–31)
Total clinician time, m, median (min–max)		33.1, 30 (8–90)

a Reversed scoring, i.e. higher score is less overwhelmed; CSQ-8, Client Satisfaction Questionnaire; CEQ, Credibility/Expectancy Questionnaire.

Textbox 1 **Participant quotes.**

**Table:** 

Regarding program content and examples (positive): *“The program motivates me to take a moment for reflection.”* *“I find [the example characters] in the program to be good reading. They offer a good variety, showing the diversity among us.”* Regarding program content and examples (negative): *“A conversation would be more beneficial than all this reading and checking boxes. It feels like a stressful, demanding chore, and it's very time-consuming.”* *“Since none of the characters has cancer, I can't completely identify with them.”* Regarding the lifefulness diary (positive): *“[The diary] provides an opportunity to reflect on what I actually do and how I actually feel, compared to my perception of what I do and how I feel.”* *“I have reached out to neighbors (the book club), sought out training opportunities (despite back injury), and splurged on something I would n-e-v-e-r have done before, [new ice-grip shoes], without feeling ashamed!”* Regarding the lifefulness diary (negative): *“There are some unclear concepts that affect the completion of the calendar.”* *“The calendar interface is somewhat challenging to navigate.”* Regarding the future need of care: *“I don't require any further care. I've had lots of therapy, and I don't want to go through that again. I want to be in charge of my own decisions.”* *“I plan to continue using the tool and will set up my own alarms and reminders.”* *“I need someone to talk to, to reflect on myself, and on living with someone whose memory function is declining.”* *“I have considerable health anxiety and am too sedentary.”*

### 3.3 Clinical outcome measures

Results regarding quality of life, as measured by the SF-36, subscales and summarized scores regarding physical health (PCS) did not change consistently to the post intervention time point and to the 3-month follow-up. The subscales and summarized scores regarding mental health (MCS) on the other hand, increased consistently to both the post intervention time point and to the 3-month follow-up (see Table 4). Subjective quality of life, as measured with EQ-5D VAS, also increased during the intervention and is presented together with the rest of the secondary outcomes in Table 5. Significant decreases over time were observed for depression, anxiety, suicidal ideation, and loneliness, even though not all participants presented with significant baseline symptoms of these problems.

**Table 4:** Quality of life measured with SF-36 subscales and raw component summaries, mean (SD) [95% CI], within-group effect sizes from baseline [95% CI], significance level (* = p < 0.05, ** = p < 0.01, *** = p < 0.001), all p-values < 0.05 are marked in bold text.

	Baseline	Mid	Post	3 month FU
n	73	70	68	62
**Physical Component raw score (PCS) (higher is better)**	70.4 (21.4) [65.4, 75.4]	67.8 (23.2) [62.2, 73.3]	70.2 (21.5) [65.0, 75.3]	67.4 (28.0) [60.3, 74.5]
Within-group effect sizes			*d* = −0.01 [−0.25, 0.23], *n.s.*	*d* = −0.19 [−0.44, 0.06], *n.s.*
**PCS Subscales (higher is better)**				
1.Physical functioning (PF)	83.5 (16.9) [79.6, 87.4]	79.4 (18.7) [74.9, 83.8]	81.8 (17.2) [77.6, 85.9]	80.5 (20.0) [75.4, 85.6]
Within-group effect sizes			*d* = −0.21 [−0.45, 0.03], *n.s.*	***d*** **=** −0.31 [−0.56, −0.05], *
2.Physical Role Functioning (RP)	70.2 (33.8) [62.3, 78.1]	71.4 (37.2) [62.6, 80.3]	77.9 (35.0) [69.5, 86.4]	72.6 (38.9) [62.7, 82.5]
Within-group effect sizes			***d*** **=** 0.25 [0.00, 0.49], *	*d* = 0.07 [−0.18, 0.32], *n.s.*
3.Bodily Pain (BP)	67.6 (24.2) [61.9, 73.3]	67.4 (24.7) [61.5, 73.3]	70.4 (24.0) [64.6, 76.2]	68.7 (25.4) [62.3, 75.2]
Within-group effect sizes			*d* = 0.12 [−0.12, 0.36], *n.s.*	*d* = −0.03 [−0.28, 0.22], *n.s.*
4. General Health Perceptions (GH)	59.7 (21.4) [54.7, 64.7]	61.9 (21.6) [56.7, 67.0]	62.6 (21.2) [57.5, 67.8]	65.3 (19.4) [60.4, 70.3]
Within-group effect sizes			*d* = 0.20 [−0.04, 0.44], *n.s.*	***d*** **=** 0.40 [0.14, 0.66], **
**Mental Component raw score (MCS) (higher is better)**	88.4 (32.4) [80.8, 95.9]	96.9 (33.4) [88.9, 105]	103 (30.8) [95.6, 111]	106 (28.1) [99.2, 113]
Within-group effect sizes			***d*** **=** 0.56 [0.30, 0.81], ***	***d*** **=** 0.60 [0.33, 0.87], ***
**MCS Subscales (higher is better)**				
1. Vitality (VT)	52.1 (21.4) [47.1, 57.1]	55.6 (23.5) [50.0, 61.3]	58.2 (22.2) [52.9, 63.6]	61.1 (20.3) [56.0, 66.3]
Within-group effect sizes			***d*** **=** 0.37 [0.12, 0.61], **	***d*** **=** 0.51 [0.24, 0.77], ***
2. Social Role Functioning (SF)	70.9 (22.7) [65.6, 76.2]	79.8 (22.3) [74.5, 85.2]	84.2 (19.5) [79.5, 88.9]	84.9 (20.9) [79.6, 90.2]
Within-group effect sizes			***d*** **=** 0.71 [0.44, 0.98], ***	***d*** **=** 0.66 [0.38, 0.93], ***
3. Emotional Role Functioning (RE)	66.2 (36.2) [57.8, 74.7]	70.5 (37.0) [61.7, 79.3]	78.9 (33.5) [70.8, 87.0]	80.6 (31.7) [72.6, 88.7]
Within-group effect sizes			***d*** **=** 0.35 [0.11, 0.60], **	***d*** **=** 0.36 [0.10, 0.61], **
4. Mental Health (MH)	64.2 (19.1) [59.7, 68.6]	69.9 (20.5) [65.0, 74.8]	72.3 (20.0) [67.5, 77.1]	73.0 (18.4) [68.4, 77.7]
Within-group effect sizes			***d*** **=** 0.57 [0.31, 0.82], ***	***d*** **=** 0.55 [0.28, 0.81], ***
**Perceived health change compared to one year prior**				
Item 2 mean (lower is better)	3.21 (0.80) [3.02, 3.39]	3.04 (0.92) [2.82, 3.26]	2.94 (0.96) [2.71, 3.17]	2.81 (0.88) [2.58, 3.03]
			***d*** **=** −0.27 [−0.51, −0.02], *	***d*** **=** −0.34 [−0.60, −0.08], **
Proportion reporting improved health, n (%)	9/73 (12%)	15/70 (21%)	19/68 (28%)	20/62 (32%)
Proportion reporting no change in health, n (%)	42/73 (58%)	32/70 (46%)	30/68 (44%)	30/62 (48%)
Proportion reporting worsened health, n (%)	22/73 (30%)	23/70 (33%)	19/68 (28%)	12/62 (19%)

**Table 5:** Secondary outcomes, mean (SD) [95% CI], proportions above cut-off, within-group effect sizes from baseline [95% CI], significance level (* = p < 0.05, ** = p < 0.01, *** = p < 0.001), all p-values < 0.05 are marked in bold text.

	Baseline	Mid	Post	3 month FU
n	73	70	67	62
**Quality of life, EQ-5D VAS**	61.7 (20.7) [56.9, 66.5]	–	70.1 (17.8) [65.8, 74.5]	–
Proportion ≥ 50, n (%)	52/73 (71%)	–	56/66 (85%)	–
Within-group effect size			***d*** **=** 0.39 [0.14, 0.64],**	–
**Depression, GDS-15**	5.3 (4.1) [4.4, 6.3]	4.2 (4.0) [3.2, 5.1]	3.3 (3.7) [2.4, 4.2]	3.3 (3.7) [2.4, 4.3]
Proportion ≥ 5, n (%)	34/73 (47%)	21/70 (30%)	18/67 (27%)	14/62 (23%)
Within-group effect sizes			***d*** **=** −0.61 [−0.86, 0.34],***	***d*** **=** −0.69 [−0.97, 0.41],***
**Anxiety, GAS-10**	6.2 (4.3) [5.2, 7.1]	4.7 (4.4) [3.6, 5.7]	3.9 (3.7) [3.0, 4.7]	3.5 (3.7) [2.6, 4.5]
Proportion ≥ 10, n (%)	14/73 (19%)	8/70 (11%)	5/67 (8%)	6/62 (10%)
Within-group effect sizes			***d*** **=** −0.58 [−0.84, 0.32],***	***d*** **=** −0.72 [−0.99, 0.44],***
**Suicidal ideation, MADRS-S item 9**	1.07 (1.21) [0.79, 1.35]	0.93 (1.33) [0.61, 1.25]	0.66 (1.07) [0.40, 0.92]	0.63 (1.04) [0.36, 0.89]
Proportion ≥ 1, n (%)	40/73 (55%)	31/70 (44%)	24/67 (36%)	21/62 (34%)
Within-group effect sizes			***d*** **=** −0.44 [−0.69, 0.19],***	***d*** **=** −0.51 [−0.77, 0.24],***
**Loneliness, DeJong**	2.81 (1.93) [2.36, 3.26]	–	1.86 (1.83) [1.41, 2.31]	–
Proportion ≥ 3, n (%)	39/73 (53%)	–	23/66 (35%)	–
Within-group effect size			***d*** **=** −0.54 [−0.79, 0.28],***	–
**Insomnia, ISI**	9.7 (5.8) [8.4, 11.0]	8.0 (5.4) [6.7, 9.3]	6.9 (5.0) [5.7, 8.1]	7.4 (5.6) [6.0, 8.8]
Proportion ≥ 15, n (%)	13/73 (18%)	9/70 (13%)	6/67 (9%)	8/62 (13%)
Within-group effect size			***d*** **=** −0.68 [−0.95, 0.42],***	***d*** **=** −0.60 [−0.86, 0.32],***
**Alcohol problem severity, AUDIT**	4.2 (4.6) [3.1, 5.3]	4.0 (4.6) [2.9, 5.1]	3.8 (4.2) [2.8, 4.8]	3.9 (4.3) [2.8, 5.0]
Proportion ≥ 5 (men) and ≥4 (women), n (%)	29/73 (40%)	25/70 (36%)	24/67 (36%)	20/62 (32%)
Within-group effect sizes			*d* = −0.17 [−0.41, 0.07], *n.s.*	***d*** **=** −0.31 [−0.56, −0.05], *
Physical activity, proportion ≥ 3 days a week of moderate or vigorous activity, n (%)	39/73 (53%)	–	44/65 (68%)	–

EQ-5D VAS, EuroQOL visual analogue scale; GDS-15, Geriatric Depression Scale; GAS-10, Geriatric Anxiety Scale; MADRS-S, Montgomery-Åsberg Depression Rating Scale–Self-Rated; DeJong, 6-item De Jong Gierveld Loneliness Scale; ISI, Insomnia Severity Index; AUDIT, Alcohol Use Disorders Identification Test.

Exploratory post-hoc analyses, presented in Supplementary Table 1, were conducted to investigate whether gender, age category (65–74, 75–85) or elevated baseline scores on either of the mental health outcomes were associated with changes in scores on the MCS. We found consistent findings across the two subgroups gender and age category, and stable decreases in mental health symptoms for participants with elevated baseline scores. Alcohol problem severity, however, remained rather constant across all time points, even for those participants with elevated baseline severity. The correlation between number of diary registrations and change in MCS to post intervention was *r* = 0.15 [−0.09, 0.37] and the correlation between number of completed modules and MCS symptom change was *r* = −0.03 [−0.27, 0.21].

## 4 Discussion

### 4.1 Discussion

This study provides a first evaluation of Lifefulness, a digital program specifically designed for promoting mental health among older adults. Feasibility and acceptability were strongly supported: most participants completed the majority of modules and registered daily, or almost daily, in the central diary tool, while reporting high satisfaction and intervention credibility. Furthermore, clinical resource use was low, with the total clinician time per participant averaging just over half an hour, distributed across three scheduled telephone interviews. This can be compared to an earlier therapist-guided digital intervention for older adults in which guidance was estimated to 100 min per participant, not counting phone interviews (Silfvernagel et al., 2018). Further, participants showed within-group reductions in symptom scores and increases in quality of life ratings across outcomes including suicidal ideation, loneliness, vitality, anxiety and depression. Exploratory subgroup analyses did not show any consistent differences in pre-post changes across sex or age brackets. While participants with higher baseline symptom levels reported larger score reductions, these differences should be interpreted with caution as they are particularly susceptible to regression to the mean. These results are encouraging, particularly as the intervention specifically targeted mental health broadly, without explicitly directing behaviour change toward a single outcome. Overall, the findings indicate that a digital mental health intervention for older adults is feasible, well-received, and associated with promising symptom trends that warrant formal testing in a randomized controlled trial.

A key finding from this feasibility trial is the clear demonstration that older adults are capable of engaging with complex digital interventions, at least in a group with, on average, a high level of education. While we prioritized a user-friendly design, the program still required participants to manage the challenges inherent in digital self-help, such as login procedures, navigating various steps and menus, scrolling to find content, and processing a substantial amount of information. The high adherence observed, where most participants completed the core behavioural exercises and logged activities in the Lifefulness diary, is particularly noteworthy. This suggests that the intervention was not limited to psychoeducation, but encouraged active engagement in behavioural exercises during the program period. Although self-reported activity logging cannot establish definitive habit formation or long-term lifestyle changes, this focus on behaviour modification is crucial, as sustained changes in daily habits increase the likelihood of durable long-term effects. Consequently, the program holds potential for long-term preventive effects against future mental health issues and related healthcare consumption, if verified in subsequent long-term trials.

### 4.2 Limitations

Limitations with this study include the short follow-up period and the lack of a quantitative measure for impact on healthcare consumption. Given the program's potential as a preventive intervention, future studies must prioritize longer follow-up periods to definitively track changes in healthcare use and associated costs. Furthermore, although we observed a qualitative breadth across our participants, the sample was predominantly female, highly educated, and likely highly motivated. This potential selection bias limits the generalizability of the findings, as high adherence and positive acceptability ratings may partly reflect this specific demographic profile. Therefore, a subsequent evaluation within a setting where the population is treatment-seeking and more heterogeneous, is essential. Another limitation is the lack of prospectively defined criteria or quantitative feasibility thresholds for behavioural engagement metrics, which future trials in this line of research should define a priori to evaluate trial progression more definitively.

### 4.3 Conclusion

This initial evaluation suggests that the intervention *Lifefulness* warrants further testing in a randomized clinical trial. Such a trial would establish the efficacy of the program, particularly its potential to increase resilience for mental health problems and function as an indicated prevention or early intervention strategy. It should be conducted in a primary care setting, utilizing a treatment-seeking population. To definitively assess potential preventive effects on the incidence of mental health disorders or effects on healthcare use/costs, longer follow-up times, ideally at least one year, are required. Furthermore, future trials will necessitate a larger sample size and should include an appropriate control group for health economic evaluations, such as care as usual. Finally, continuous refinement of the current digital intervention, based on ongoing stakeholder feedback, should be an integrated component of all future research efforts.

## Declaration of Generative AI and AI-assisted technologies in the writing process

During the preparation and revision of this work, the authors used Gemini to improve language clarity. The authors reviewed and edited the output as needed and take full responsibility for the content of the published article.

## Funding

The study is part of a larger international initiative focusing on improving mental health for older adults worldwide, with parallel studies currently being conducted in both Sweden and India. This study was funded by 10.13039/501100006636FORTE, grant number 2022-01155. The funder has no role in the design of this study, and was not involved in data collection, data analysis, and decision to submit results.

## Declaration of competing interest

The authors declare none.

## Data Availability

The de-identified quantitative datasets generated and analysed during the current study can be made available to qualified researchers for academic, non-commercial research purposes (e.g., data pooling or meta-analyses). Access is governed by the ethical approval granted by the Swedish Ethical Review Authority (Dnr 2024-00363-01, amended 2025-01276-02) and is subject to compliance with applicable privacy regulations, participant consent limitations, and the establishment of a formal Data Transfer Agreement. Requests for access to the quantitative data should be directed to the Karolinska Institutet Research Data Office (rdo@ki.se), with the corresponding author (martin.kraepelien@ki.se) available as the scientific point of contact. Qualitative transcripts are not available to protect participant privacy and confidentiality.

## Appendix A Supplementary data

Supplementary Table 1 Exploratory data on SF-36 Mental Health Component outcomes stratified by gender and age, as well as mental health outcomes specifically for participants who scored above the relevant clinical cut-offs at baseline, mean (SD) [95% CI].
