# Supplementary material for: Lifefulness - feasibility and acceptability of a self-guided digital intervention promoting mental health among older adults in Sweden
Source: Internet Interv. 2026 Sep 28;46:101005. doi: 10.1016/j.invent.2026.101005 (PMC13651674; doi:10.1016/j.invent.2026.101005)
Supplement: Supplementary file 1 [file mmc1.docx]

**Supplementary Table 1.** Exploratory data on SF-36 Mental Health Component outcomes stratified by gender and age, as well asmental health outcomes specifically for participants who scored above the relevant clinical cut-offs at baseline, mean (SD) [95% CI].

|  | Baseline | Mid | Post | 3 month FU |
| --- | --- | --- | --- | --- |
| **SF-36 Mental Component raw score (MCS)** |  |  |  |  |
| Females, n=52 | 88.9 (31.4) [80.1, 97.6] | 96.6 (33.3) [87.1, 106] | 104 (28.0) [96.3, 112] | 109 (24.7) [101, 116] |
| Within-group effect sizes |  |  | ***d*=0.49 [0.19, 0.79], ****** | ***d*=0.57 [0.25, 0.89], ******* |
| Males, n=21 | 87.2 (35.5) [71.1, 103] | 97.6 (34.7) [81.4, 114] | 99.8 (37.7) [81.7, 118] | 101 (35.5) [83.8, 119] |
| Within-group effect sizes |  |  | ***d*=0.93 [0.38, 1.46], ******* | ***d*=0.72 [0.19, 1.23], ****** |
| Age 65-74, n=56 | 89.0 (31.7) [80.5, 97.5] | 98.7 (33.4) [89.6, 108] | 104 (31.1) [95.1, 112] | 107 (29.1) [98.7, 116] |
| Within-group effect sizes |  |  | ***d*=0.56 [0.27, 0.85], ******* | ***d*=0.62 [0.30, 0.92], ******* |
| Age 75-85, n=17 | 86.3 (35.4) [68.1, 104] | 90.8 (34.0) [72.7, 109] | 101 (30.8) [84.5, 117] | 104 (25.3) [89.0, 118] |
| Within-group effect sizes |  |  | *d*=0.52 [-0.01, 1.03], *n.s.* | *d*=0.55 [-0.03, 1.10], *n.s.* |
| **Depression, GDS-15,** participants ≥5 at baseline, n=34 | 8.9 (3.1) [7.9, 10.0] | 6.5 (4.1) [5.1, 8.0] | 5.2 (4.0) [3.8, 6.7] | 5.3 (4.3) [3.7, 6.9] |
| Within-group effect sizes |  |  | ***d*=-1.04 [-1.47, -0.60], ******* | ***d*=-1.04 [-1.48, -0.59], ******* |
| **Anxiety, GAS-10,** participants ≥10 at baseline, n=14 | 13.2 (3.0) [11.5, 15.0] | 9.4 (5.1) [6.3, 12.4] | 6.6 (4.2) [3.9, 9.2] | 7.6 (5.5) [3.8, 11.3] |
| Within-group effect sizes |  |  | ***d*=-2.09 [-3.11, -1.05], ******* | ***d*=-0.98 [-1.69, -0.24], ****** |
| **Suicidal ideation,** MADRS-S item 9, participants ≥1 at baseline, n=40 | 1.95 (0.96) [1.64, 2.26] | 1.62 (1.44) [1.15, 2.08] | 1.11 (1.24) [0.69, 1.52] | 1.11 (1.18) [0.71, 1.52] |
| Within-group effect sizes |  |  | ***d*=-0.77 [-1.14, -0.40], ******* | ***d*=-0.76 [-1.13, -0.37], ******* |
| **Loneliness,** DeJong, participants ≥3 at baseline, n=39 | 4.33 (1.18) [3.95, 4.71] | - | 2.74 (1.78) [2.11, 3.36] | - |
| Within-group effect size |  |  | ***d*=-0.96 [-1.36, -0.55], ******* | - |
| **Insomnia, ISI,** participants ≥15 at baseline, n=13 | 18.9 (3.9) [16.6, 21.3] | 14.2 (5.2) [11.1, 17.3] | 12.3 (5.8) [8.8, 15.8] | 13.8 (6.0) [10.1, 17.6] |
| Within-group effect sizes |  |  | ***d*=-1.09 [-1.77, -0.38], ****** | ***d*=-0.76 [-1.40, -0.10], ***** |
| **Alcohol problem severity**, AUDIT, participants ≥5 (men) and ≥4 (women) at baseline, n=29 | 8.1 (5.0) [6.3, 10.0] | 7.3 (5.4) [5.2, 9.3] | 7.0 (4.7) [5.1, 8.9] | 7.1 (5.0) [5.1, 9.1] |
| Within-group effect sizes |  |  | *d*=-0.35 [-0.74, 0.05], *n.s.* | ***d*=-0.47 [-0.87, 0.06], ***** |
